# Metastatic Male Ductal Breast Cancer Mimicking Obstructing Primary Colon Cancer

**Published:** 2010-03

**Authors:** Issam Koleilat, Anil Syal, Muhammad Hena

**Affiliations:** *Division of Surgical Oncology, Department of General Surgery, Albany Medical Center Hospital, 43 New Scotland Avenue, Albany, New York, USA*

**Keywords:** breast, cancer, colon, male, metastasis

## Abstract

Male breast cancer comprises only about 1% of all breast cancers. Commonly, sites of metastases include the central nervous system, lungs, bones, and even liver. In females, extrahepatic gastrointestinal metastases are unusual but have been reported with various clinical presentations. We are reporting the first case of a male patient with a history of ductal breast carcinoma that developed colonic metastasis and presented with mechanical large bowel obstruction masquerading as primary colon cancer.

## INTRODUCTION

Breast cancer is the most common cancer afflicting women. Survival is improved with early diagnosis and treatment. The risk of metastatic disease increases with increased patient survival after initial diagnosis. Overall, only 1% of all breast cancers occur in men. Almost always the pathology in male breast cancer is ductal carcinoma. Typically, breast carcinoma metastasizes to the bone, brain, and lung. Very rarely are metastases found in the extrahepatic gastrointestinal system and have been reported with various clinical presentations in females. It is herein described the first case of a 54 year-old gentleman who developed large bowel obstruction secondary to a breast cancer metastasis years after diagnosis.

## CASE

JW is a 54 year-old gentleman who presented to the emergency department with a one-week history of progressively worsening diffuse abdominal pain that began in the periumbilical region and progressed to the left lower quadrant (LLQ). The pain was initially intermittent and by the time of presentation had become intractable and severe. He reported mild alleviation by lying left laterally recumbent. His last bowel movement was four days prior to presentation with normal color and consistency. He did not have any relief with the use of glycerin suppository or fleet enemas. The pain was worse with movement. He denied any melena or hematochezia. He did confirm increasing nausea in the day prior to admission and two episodes of emesis. He has never had any episode like this. He denied fevers, chills, lightheadedness, and dizziness.

JW’s past medical history is significant for male ductal breast cancer initially diagnosed in 1996. He underwent right modified radical mastectomy and axillary lymph node dissection that same year for T2N1aM0 (grade 1) disease. His pathology initially returned ER+/PR+ cells. He underwent adjuvant chemotherapy with Adriamycin and Cytoxan for four cycles followed by Tamoxifen for five years. He had two chest wall recurrences in 2001 and in 2003, for which he underwent radiation and chemotherapy and was being maintained on Arimedex. Early in 2008 he had a PET scan that revealed mild metabolic activity in the gastric cardia and fundus, most likely gastritis or peptic ulcer disease.

He also has a protein C deficiency diagnosed after a DVT in the left arm secondary to a PICC line. He denied any history of abdominal surgeries. He is followed by a gastroenterologist for evaluation of ulcer-like symptoms. He had an esophagogastroduodenoscopy with routine colonoscopy scheduled a month away.

Medications on presentation included Coumadin and Arimidex. He has allergies to shellfish and sulfa drugs. He is married and a truck driver. He formerly smoked half a pack of cigarettes per day for 12 years, stopped, and recently had restarted. There is rare alcohol usage and he denies any toxin or chemical exposure. He denied any illicit drug use.

His mother was deceased at 74 years of age secondary to lung cancer. His father was deceased at 80 of an unknown malignancy thought to be lung. He has two brothers and four children, all alive and well.

On examination, he was afebrile and with normal vital signs. He was in obvious pain. The abdomen was tender to palpation, especially in the LLQ, soft and with a notable fullness, especially in the LLQ. There was no rebound or guarding. Digital rectal exam revealed an empty vault without stool or masses and was guaiac negative. His physical exam was otherwise unremarkable.

Laboratory results were overall unremarkable. He had no leukocytosis. His electrolytes and liver function tests were within normal limits.

**Figure 1:**
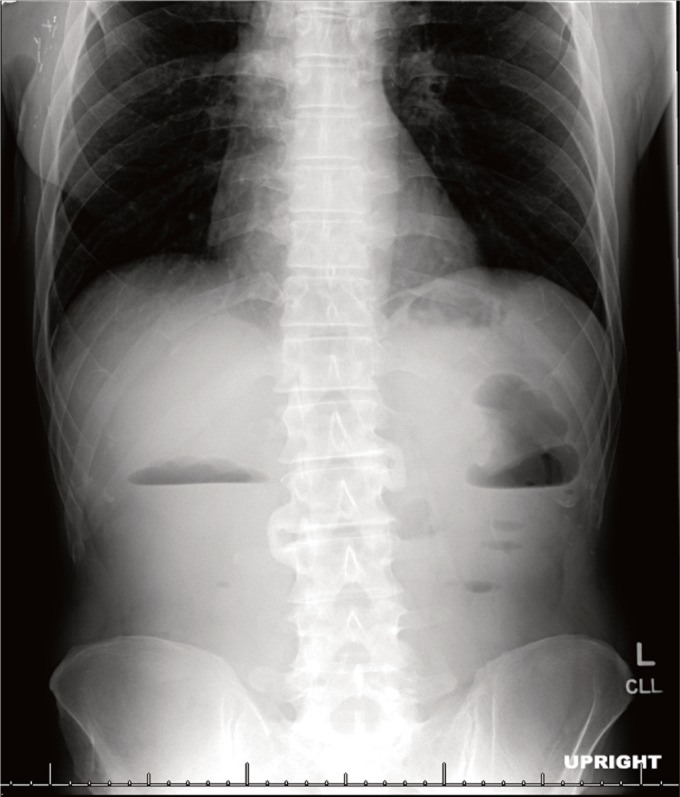
Abdominal x-ray on presentation revealing air-fluid levels.

Abdominal x-ray revealed air and stool to the level of the rectum. There was an air fluid level on the upright view in the region of the hepatic and splenic flexures (Figure 1). The small bowel gas pattern was essentially unremarkable.

**Figure 2:**
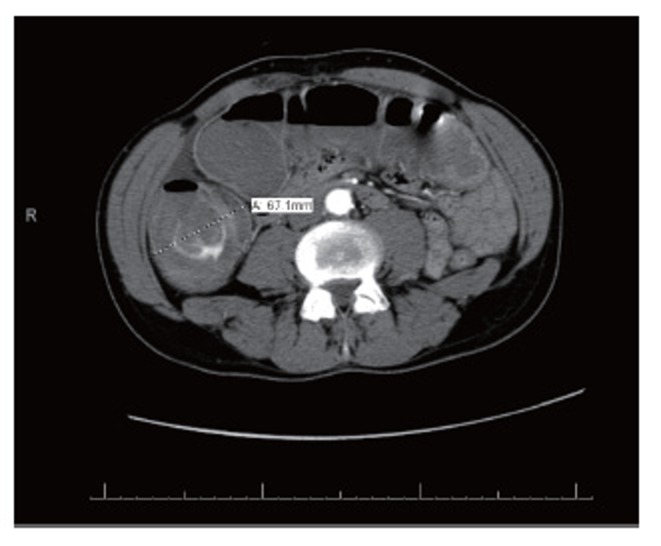
CT with oral and IV contrast on presentation. Cecum dilated to 6.7 cm.

**Figure 3:**
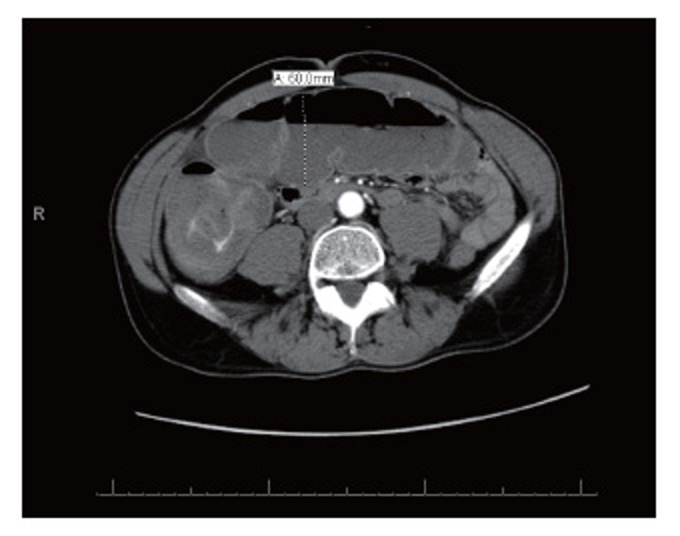
CT with oral and IV contrast on presentation. Transverse colon dilated to 6 cm with collapse of large bowel distal to splenic flexure. There is no obvious identifiable mass.

Computed Tomography (CT) with oral and intravenous contrast revealed dilatation of a fluid-filled cecum, ascending and transverse colon. The maximal diameter was 6.0 cm. A transition point was noted at the splenic flexure with distal collapsed bowel. This was suggestive of a large bowel obstruction (Figures 2 and 3). No obvious obstructing mass could be identified.

A gastrograffin enema revealed a persistent deformity of the bowel loop seen in the region of the splenic flexure. Contrast did pass and filling of the ascending colon with contrast was seen. The caliber of the ascending colon was in the upper limits of normal. There was no extravasation of contrast or evidence of perforation.

**Figure 4:**
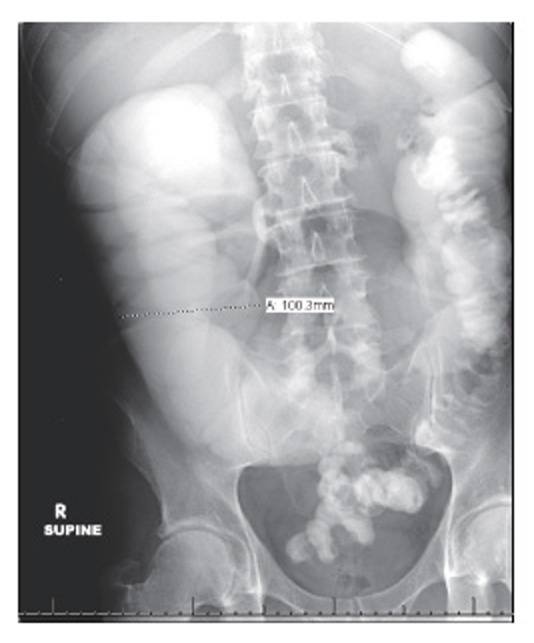
Follow-up abdominal roentgenogram after barium enema revealed retained contrast with increasingly dilated proximal colon to 10 cm.

The patient began to improve clinically but continued to have high nasogastric tube output. Due to the lack of bowel prep and the suspected obstruction, colonoscopy was avoided. A follow-up post-enema abdominal x-ray was obtained to evaluate for passage of contrast. This study found contrast still retained in the large colon with dilated loops of large bowel increased in size to 10 cm (Figure 4). Air-fluid levels at the hepatic and splenic flexures were identified.

On hospital day three, he underwent an exploratory laparotomy. Intraoperatively, he was found to have a splenic flexure mass and stricture, dilated transverse and right colon, tumor implant in right colon, multiple other colonic implants, and ascites. This was suspicious for primary colorectal tumor and thus he underwent subtotal colectomy with end-ileostomy.

Postoperatively, JW did well. His hypercoagulability had been managed with therapeutic anticoagulation. He had no postoperative complications, his condition markedly improved and he was discharged home on post-operative day six.

**Figure 5:**
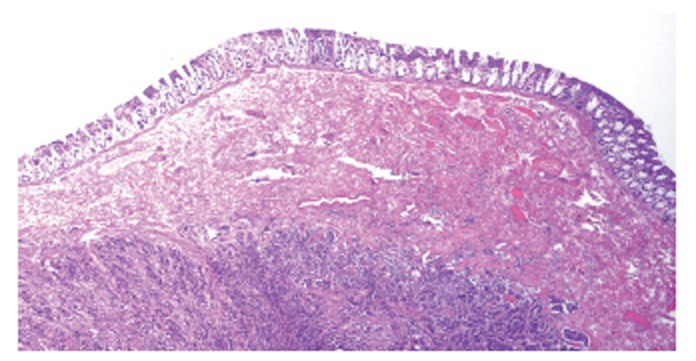
Segment of Proximal Colon Demonstrating Metastatic Adenocarcinoma of Male Breast Origin. Low magnification image demonstrating intramural tumor mass. Note absence of extension to the mucosal surface (hematoxylin and eosin × 40).

**Figure 6:**
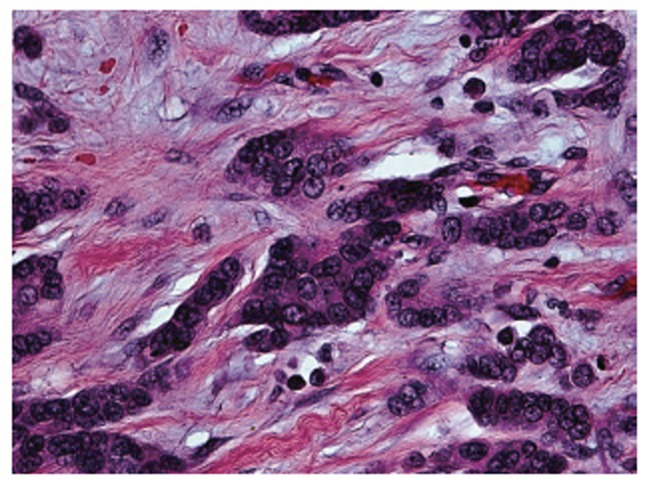
Segment of Proximal Colon Demonstrating Metastatic Adenocarcinoma of Male Breast Origin. High magnification image demonstrating an adenocarcinoma featuring infiltrating cords and single cell columns with occasional tubular lumina (hematoxylin and eosin × 400).

The final pathology report revealed benign ascitic fluid with reactive mesothelial cells and chronic inflammation. The mass was found to have pathology consistent with metastatic carcinoma of male breast origin. Multiple serosal and intramural nodules of infiltrating ductal-type adenocarcinoma were found (Figure 5). The specimen did show single cell columns or “indian filing” (Figure 6).

Both ER and PR were strongly positive and Her-2/neu was negative by FISH. The proximal and distal specimen margins were negative for malignancy. The appendiceal serosa also demonstrated metastatic carcinoma. Multiple nodules of carcinoma within the intestinal serosa and mesentery were found that were consistent with replaced lymph nodes, although residual lymph node parenchyma was not identified in these sites.

JW is doing well now at ten months post-operatively, having undergone PET scan three weeks after discharge with again only increased activity in the gastric cardia and fundus reported as most likely gastritis or peptic ulcer disease. He also had ileostomy takedown and chemotherapy with Taxotere and Xeloda.

## DISCUSSION

Male breast cancer of ductal histopathology metastatic to the large bowel causing large bowel obstruction and imitating primary colon cancer has not yet been reported to our knowledge. However, in females, the incidence of extrahepatic gastrointestinal metastases from breast carcinoma is reported in autopsy studies as 6 – 18% (1). Although it was initially thought that gastric lesions may be slightly more frequent than lower gastrointestinal lesions (2), new data suggests that colorectal metastases may outnumber gastric metastases (45% versus 28%, respectively) (3). The metastatic lesion may be the initial presentation of breast cancer (4) or may present as remotely as thirty years after initial diagnosis (2). Clinically apparent manifestations have been reported in less than 1% of cases (5). The incidence is likely underestimated as a result of the nonspecificity of presenting symptoms, patient death secondary to other etiologies or even clinical and radiologic evidence suggesting a de novo primary rather than a metastatic lesion (6). Additionally, a long latency of colonic lesions, difficulty screening and even attribution of symptoms to chemotherapy sequelae or paraneoplastic syndromes may contribute to this problem (7).

Interestingly, there seems to be a preference of infiltrating lobular carcinoma for gastrointestinal metastases despite the higher prevalence of infiltrating ductal carcinoma (8). In fact, the likelihood of gastrointestinal metastases of infiltrating lobular carcinoma may be about three to twenty times that of ductal carcinoma (9, 10). Frequently, the serosa, muscularis, and even submucosa can be infiltrated by small cells with round, monomorphic nuclei and vacuolated cytoplasm. When this occurs, they are typically arranged in single-cell cords or columns, named “indian files.” This pattern of infiltration may provoke such an immune response that surrounding tissue undergoes a severe fibrotic reaction.

Breast carcinoma metastatic to the gastrointestinal tract, although uncommon, should thus be part of the differential diagnosis for those presenting with GI complaints (early satiety, hematochezia, anorexia, bloating, obstipation, etc.) and a history of breast cancer (2). All too frequently, presenting symptoms are attributed to already known disseminated disease or sequelae of chemotherapy (6). Although a new primary colorectal tumor is more common that a metastatic breast cancer lesion (11, 12), it is quite important to differentiate so that appropriate surgical and medical therapy may be instituted (13, 14).

There is not yet a consensus as to the most appropriate therapy (15, 16). Most recommendations include palliative surgery for symptomatic disease and systemic antineoplastic medical therapy (14). The median survival after diagnosis is 28 months with surgical intervention having no effect on overall survival (3).

Interestingly, this is the first report of colonic metastases from a primary male breast adenocarcinoma of ductal histology. Additionally, although described in females in sporadic case reports in the literature, this is the first report to the knowledge of the authors of a large bowel obstruction from metastatic breast carcinoma in a male patient mimicking colon primary.
